# Awareness Regarding Diabetic Retinopathy Among Adult Diabetic Patients in Al Qunfudah District

**DOI:** 10.7759/cureus.31773

**Published:** 2022-11-22

**Authors:** Safa H Alkalash, Amal A Alrizqi, Amnah I Al Kenani, Shroog M Alessi, Rahaf A Alqozi, Amal A Alamri, Reda Goweda

**Affiliations:** 1 Community Medicine and Health Care, Umm Al-Qura University, Al Qunfudah, SAU; 2 Family Medicine, Menoufia University, Shibin Al Kawm, EGY; 3 Medical Student, Umm Al-Qura University, Al Qunfudah, SAU; 4 Family Medicine, Umm Al-Qura University, Makkah, SAU; 5 Family Medicine, Suez Canal University, Ismailia, EGY

**Keywords:** public health, blindness, complications, diabetic retinopathy, diabetes mellitus

## Abstract

Background

Diabetes mellitus is a major public health problem worldwide. Diabetic patients may suffer many complications including diabetic retinopathy, which can lead to blindness if left undiagnosed and untreated.

Methodology

A cross-sectional analytical study was conducted among a sample of 251 adult diabetic patients in the Al Qunfudah district. A self-administrated questionnaire was used for data collection and consisted of the following three sections: sociodemographic data, diabetes mellitus, and diabetic retinopathy-related data.

Results

Out of the 251 participants, 68.5% recognized that diabetes mellitus can cause eye disorders, and 72.5% of the participants knew that regular eye examinations are recommended for diabetic patients. Adequate knowledge score was observed among diabetic patients aged 25-49 years (52.6%), with a university education (53.5%), with a history of diabetes for 10 years and more (60.5%), those on insulin therapy (55.3%), those adherent to their regular treatment (92.1%), and those getting their information from doctors (73.7%).

Conclusions

Diabetic patients had average knowledge about diabetic retinopathy but little awareness regarding the frequency of regular eye examinations and the physicians who should conduct them. Intensive health education concerning diabetic retinopathy should be conducted for diabetic patients and their caregivers to improve their perception and compliance with eye care and prevent visual damage in the Al Qunfudhah district.

## Introduction

In 2014, the global prevalence of diabetes mellitus was estimated to be 9% among men and 7.9% in women, substantially doubling from the 1980 statistics of 4.3% and 5% in men and women, respectively [1]. In Saudi Arabia, some studies have reported a 30% prevalence of diabetes mellitus among the Saudi population [2]. This prevalence is linked to the recent economic boom in the country, which has resulted in significant lifestyle changes, with poor eating habits and sedentary lifestyles becoming the norm [2]. Diabetes mellitus is one of the most common metabolic disorders caused by either defective insulin secretion by pancreatic β-cells or the inability of insulin-sensitive tissues to respond appropriately to insulin [3].

Diabetic mellitus induces pathological changes in blood vessels, particularly in the retinal microvasculature, resulting in organ and tissue damage among approximately one-third to one-half of diabetic patients [4]. Diabetic retinopathy is the leading cause of visual impairment worldwide. Out of 285 million diabetic patients worldwide, about one-third have signs of diabetic retinopathy and another one-third have vision-threatening diabetic retinopathy, including diabetic macular edema [5]. Several studies have examined the risk factors of diabetic retinopathy and found both modifiable and non-modifiable risk variables for its presence and severity. Risk factors that can be corrected include elevated blood sugar, blood pressure, serum lipids, and smoking. However, non-modifiable factors include disease duration, age, genetic predisposition, and ethnicity. Other risk factors are pregnancy, the number of microaneurysms in one eye, the microaneurysm formation rate, and the presence of diabetic retinopathy in the second eye [6].

Awareness of diabetic patients and their caregivers regarding the management of diabetes mellitus and the importance of regular health follow-ups is a fundamental step toward improving prognosis and limiting its consequences such as diabetic retinopathy. Therefore, many studies have been conducted to evaluate the knowledge among diabetic patients and their caregivers, with most studies concluding insufficient to average awareness levels. A study on awareness, knowledge, and practices related to diabetic retinopathy among diabetic patients in primary healthcare centers in Riyadh, Saudi Arabia concluded that despite 61% of patients having good knowledge, less than half (45%) had their eyes checked within one year [7].

In Saudi Arabia, the Ministry of Health in Jeddah conducted a cross-sectional survey among diabetic patients who visited primary healthcare centers to assess their level of awareness regarding diabetic retinopathy and its related risk factors. About 82.6% of the patients were aware that diabetes mellitus could affect their eyes, and they listed physicians, ophthalmologists, television, and family members as common sources of information on this topic. About 36% of the patients reported that their doctors had not informed them about it [8]. Measuring the awareness level among diabetic patients regarding diabetic retinopathy and its associated factors is necessary to reduce its incidence. Therefore, this study aimed to assess the awareness of diabetic patients about diabetic retinopathy in the Al Qunfudah district.

## Materials and methods

An analytical, cross-sectional survey-based study was conducted over a 12-month period from November 2021 to November 2022 in Al Qunfudah District, Saudi Arabia, located in the Tihamah region on the Red Sea coast. Its population is 300,516, representing the fourth largest in Makkah Province. With an estimated area of 5,195 km², Al Qunfudah occupies about 3.7% of the regional area. Al Qunfudah was discovered in 709 Hijri according to ancient sources. It is considered a remote area as it is about 400 km away from Jeddah and Mecca Almokarama, the nearest two big cities. Al Qunfudah district has about 20 primary healthcare centers (PHCs) run by the Ministry of Health. The study was conducted among diabetic patients attending four PHCs (Khalidiya, Al Sharqiya, Uhud Bani Zaid, and Al Quoz). All male and female diabetic patients aged 18 years and older attending the four PHCs during the period of data collection were considered eligible for inclusion in this study.

Sample size

The sample size was calculated using Epi-info version 7 based on the total number of diabetics in the Al Qunfudah district (34,550). The prevalence of diabetic retinopathy in Saudi Arabia is 36.4% [9]. At a confidence interval of 90% and a 5% margin of error, the sample size was calculated at 251.

Data collection

A simple interview was conducted for diabetic patients in the waiting rooms of PHCs and they were invited verbally to participate in this study and answer questions about diabetes mellitus and diabetic retinopathy through an online survey that was created on a Google Forms application which was sent to them via their WhatsApp applications. Furthermore, they were asked to share the survey link with other diabetic patients whether in their families or friends to increase the response rate through a snowball sampling technique.

A total of 319 questionnaires were collected. Incomplete questionnaires (n = 68) were discarded, with the final valid and complete questionnaires at 251.

Data collection tools

The online questionnaire was designed by the study researchers and comprised 19 questions subdivided into four domains. The first domain involved one question to obtain patients’ written consent for participating in this study. The second domain included five questions regarding participants’ sociodemographic data, such as age, sex, nationality, marital status, and educational level. The third domain comprised five questions about their history of diabetes mellitus (duration, type, and type of treatments, regular follow-up, monitoring, and the source of information). The fourth domain included seven questions related to diabetic retinopathy (patients’ knowledge regarding the possibility of developing diabetic retinopathy in the presence of uncontrolled diabetes mellitus, previous diagnosis of diabetic retinopathy, regular follow-up, and examination of their eyes) [10]. Table 1 present the three-point scale used to grade knowledge.

**Table 1:** The mean score of knowledge. The mean score was 0.63 which indicates a 63.0% level of knowledge about diabetic retinopathy.

Score	Knowledge level	Mean score
3	Adequate knowledge	0.76–1.00
2	Moderately adequate knowledge	0.51–0.75
1	Inadequate knowledge	0.00–0.50

A pilot study was done on 10% of the study sample (26 participants). They were from the same location to estimate the clarity and applicability of the study tool and to recognize the obstacles that may face data collection, as well as possible actions to overcome. All obtained data from the pilot study were used as a guide by the study researchers and were excluded from the main study results. To confirm the reliability of the questionnaire scores, the test-retest technique was used.

Data analysis

The data analysis of this study was done in two stages. The first stage included a descriptive analysis where numerical variables were reported as means and standard deviations while categorical variables were described using frequencies and percentages. The mean knowledge score was computed using a Likert scale analysis. The second stage included hypothesis testing using the chi-square test and the likelihood chi-square test using SPSS Statistics version 25.0 (IBM Corp., Armonk, NY, USA).

**Figure 1:**
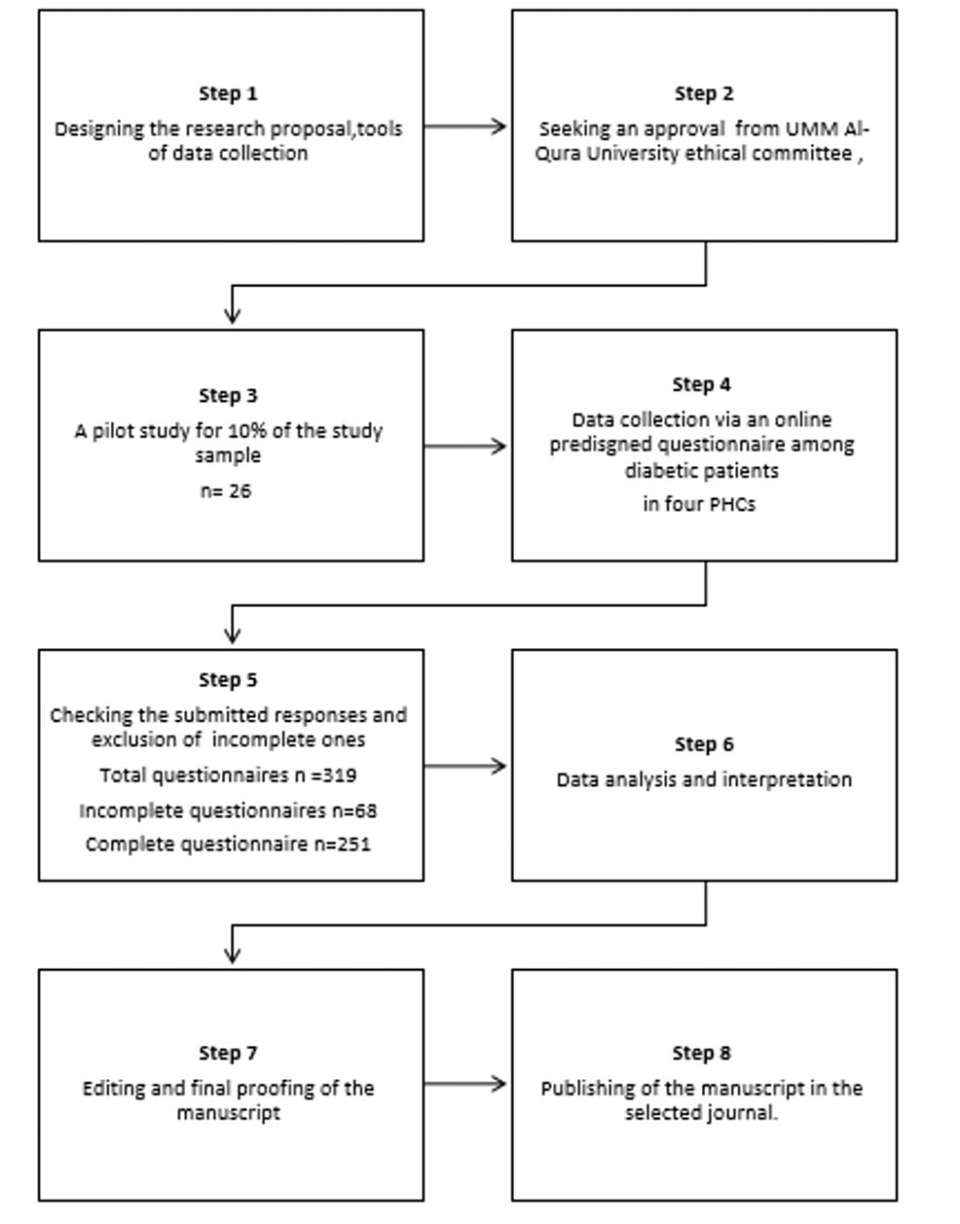
Consort diagram of the study framework.

## Results

Of the 251 participants, 151 (60.2%) were females, 238 (94.8%) were Saudis, and 139 (55.4%) were married (Table 2). About 114 (45.4%) patients had an age range from 25 to 49 years. University education was predominant among the studied group with 101 (40.2%) patients. Furthermore, a maximum of 94 (37.5%) patients had been diabetics for more than 10 years. Moreover, 120 (47.8%) participants were taking oral antidiabetic drugs, and 160 (63.7%) were adherent to a certain diabetic control diet. Further, 217 (86.5%) participants were compliant with their regular medications, and 141 (56.2%) received medical information from their doctors.

**Table 2:** Demographic data of the studied group. DM = diabetes mellitus

Characteristics	(n = 251)	(%)
Gender		
Male	100	39.8
Female	151	60.2
Nationality		
Saudi	238	94.8
Non-Saudi	13	5.2
Social status		
Unmarried	66	26.3
Married	139	55.4
Widow/divorced	46	18.3
Age		
18–24 years	31	12.4
25–49 years	114	45.4
50 years and above	106	42.2
Education level		
Reading and writing	74	29.5
University level	101	40.2
Below university level	76	30.3
Duration of DM		
Less than 5 years	92	36.7
Five to 10 years	65	25.9
More than 10 years	94	37.5
Management of DM		
Pills	120	47.8
Insulin needles	78	31.1
Both together	53	21.1
Commitment to a certain diet for DM		
No	91	36.3
Yes	160	63.7
Adherence to antidiabetic drugs		
No	34	13.5
Yes	217	86.5
Source of information about DM		
Doctor	141	56.2
Friends and family	39	15.5
Another diabetic patient	53	21.1
The media	18	7.2

More than two-thirds of the diabetic patients (68.5%) were aware that diabetes could cause eye disorders, 182 (72.5%) knew that they should conduct a regular eye examination, and 162 (64.5%) denied the need to visit an ophthalmologist whenever their blood glucose level was controlled. Most patients (82.9%) agreed that timely treatment could prevent or delay eye damage (Table 3).

**Table 3:** Knowledge about diabetic retinopathy. All values are presented as numbers and percentages. *: True answers.

Question	(n = 251)	(%)
Diabetes mellitus can cause eye problems		
No	79	31.5
Yes*	172	68.5
Diabetics should conduct regular eye examinations		
No	69	27.5
Yes*	182	72.5
There is no need to visit an ophthalmologist when diabetics have controlled blood glucose levels		
No*	89	35.5
Yes	162	64.5
Timely treatment can prevent/delay damage due to diabetes in the eyes		
No	43	17.1
Yes*	208	82.9

Table 4 presents the choice of healthcare professional to examine the eye. Overall, 229 (91.2%) patients reported an optometrist, while 149 (59.4%) and 120 (47.8%) patients reported an ophthalmologist and a general practitioner, respectively.

**Table 4:** Knowledge about physicians consulted in the event of an eye problem. All values are presented as numbers and percentages. *: True answers.

Physicians consulted	(n = 251)	(%)
Ophthalmologist (n = 251)		
No*	102	40.6
Yes	149	59.4
Any specialist (non-ophthalmologist) (n = 251)		
No*	201	80.1
Yes	50	19.9
Optometrist (n = 251)		
No	22	8.8
Yes*	229	91.2
General practitioner (n = 251)		
No	131	52.2
Yes*	120	47.8

Table 5 describes patients’ knowledge about available treatments for diabetic retinopathy. Overall, 68.1% and 69.7% of patients documented that lifestyle modifications and surgical procedures are treatment approaches for diabetic retinopathy, and (78.5%) agreed that diabetes control can be helpful for the treatment of diabetic retinopathy. Regarding the frequency of eye examinations, 43.0% reported that they went for only one visit, while 36.3% underwent yearly ocular examinations.

**Table 5:** Knowledge about available treatments for diabetic retinopathy.

	(n = 251)	(%)
No treatment available		
No*	176	70.1
Yes	75	29.9
Modification of lifestyle		
No	80	31.9
Yes*	171	68.1
Control of diabetes		
No	54	21.5
Yes*	197	78.5
Surgical procedures		
No	76	30.3
Yes*	175	69.7
Only medication		
No*	127	50.6
Yes	124	49.4
Alternative medical therapies		
No	127	50.6
Yes*	124	49.4
Frequency of eye examination		
Monthly	10	4.0
Once in six months	42	16.7
Yearly*	91	36.3
This is the first time	108	43.0

Inadequate knowledge was noticed regarding seeing an ophthalmologist when diabetes was controlled (mean = 0.35) or regular eye examination by an ophthalmologist (mean = 0.36) (Table 6). Additionally, poor knowledge was observed regarding the role of both ophthalmologists and general practitioners in performing follow-up eye examinations (mean = 0.41 and 0.48, respectively). On the other hand, there was moderately adequate knowledge (mean = 0.68) about the importance of lifestyle modifications in preventing diabetic retinopathy.

**Table 6:** Participant’s level of knowledge about diabetic retinopathy. Mean score = 0.63

Statement	Mean	SD	Rank	Level of knowledge
Diabetes mellitus can cause eye problems	0.69	0.465	8	Moderately adequate knowledge
Diabetics should conduct regular eye examinations	0.73	0.447	5	Moderately adequate knowledge
There is no need to visit an ophthalmologist when diabetics have controlled blood glucose levels	0.35	0.479	15	Inadequate knowledge
Timely treatment can prevent/delay damage due to diabetes in the eyes	0.83	0.378	2	Adequate knowledge
Physicians consulted in the event of an eye problem				
5.1 Ophthalmologist	0.41	0.492	13	Inadequate knowledge
5.2 Any specialist (non-ophthalmologist)	0.80	0.400	3	Adequate knowledge
5.3 Optometrist	0.91	0.283	1	Adequate knowledge
5.4 General practitioner	0.48	0.501	12	Inadequate knowledge
Knowledge of available treatments for diabetic retinopathy				
6.1 No treatment available	0.70	0.459	6	Moderately adequate knowledge
6.2 Modification of lifestyle	0.68	0.467	9	Moderately adequate knowledge
6.3 Control of diabetes	0.78	0.412	4	Adequate knowledge
6.4 Surgical procedures	0.70	0.460	7	Moderately adequate knowledge
6.5 Only medication	0.51	0.501	10	Moderately adequate knowledge
6.6 Alternative medical therapies	0.49	0.501	11	Inadequate knowledge
Frequency of eye examinations	0.36	0.482	14	Inadequate knowledge

Adequate knowledge score was observed significantly among diabetic patients who were 25-49 years old (p = 0.047), had a university education (p = 0.017), had a history of diabetes for 10 years and more (p = 0.018), were on insulin therapy (p = 0.000), were adherent to their regular treatment (p = 0.022), and those have received their information from doctors (p = 0.032) (Table 7).

**Table 7:** The relationship between participants’ sociodemographic data and level of knowledge about diabetic retinopathy. DM = diabetes mellitus *: Association found at a 0.05 level of significance.

Demographics	Level of knowledge			P-value
	Inadequate knowledge n (%)	Moderately adequate knowledge n (%)	Adequate knowledge n (%)	
Gender				
Male	20 (38.5)	64 (39.8)	16 (42.1)	0.940
Female	32 (61.5)	97 (60.2)	22 (57.9)	
Age				
18–24 years	7 (13.5)	16 (9.9)	8 (21.1)	0.047*
25–49 years	17 (32.7)	77 (47.8)	20 (52.6)	
50 years and above	28 (53.8)	68 (42.2)	10 (26.3)	
Nationality				
Saudi	49 (94.2)	153 (95.0)	36 (94.7)	0.975
Non-Saudi	3 (5.8)	8 (5.0)	2 (5.3)	
Social status				
Unmarried	13 (25.0)	37 (23.0)	16 (42.1)	0.062
Married	27 (51.9)	92 (57.1)	20 (52.6)	
Widow/divorced	12 (23.1)	32 (19.9)	2 (5.3)	
Education level				
Reading and writing	18 (34.6)	52 (32.3)	4 (10.5)	0.017*
University level	14 (26.9)	66 (41.0)	21 (55.3)	
Below university level	20 (38.5)	43 (26.7)	13 (34.2)	
Duration of DM				
Less than five years	22 (42.3)	64 (39.8)	6 (15.8)	0.018*
Five to 10 years	13 (25.0)	43 (26.7)	9 (23.7)	
More than 10 years	17 (32.7)	54 (33.5)	23 (60.5)	
Management of DM				
Pills	27 (51.9)	82 (50.9)	11 (28.9)	0.000*
Insulin needles	7 (13.5)	50 (31.1)	21 (55.3)	
Both together	18 (34.6)	29 (18.0)	6 (15.8)	
Commitment to a certain diet for DM				
No	18 (34.6)	61 (37.9)	12 (31.6)	0.739
Yes	34 (65.4)	100 (62.1)	26 (68.4)	
Adherence to antidiabetic drugs				
No	13 (25.0)	18 (11.2)	3 (7.9)	0.022*
Yes	39 (75.0)	143 (88.8)	35 (92.1)	
Source of information about DM				
Doctor	21 (40.4)	92 (57.1)	28 (73.7)	0.032*
Friends and family	13 (25.0)	22 (13.7)	4 (10.5)	
Another diabetic patient	15 (28.8)	35 (21.7)	3 (7.9)	
The media	3 (5.8)	12 (7.5)	3 (7.9)	

## Discussion

Diabetes mellitus is one of the most widespread diseases of the 21st century. Diabetes and other such chronic diseases are already wreaking havoc on people’s health and wealth. Due to a lack of awareness about their disease, patients with diabetes mellitus may experience a variety of complications. Diabetic retinopathy is one of these complications which is a leading cause of blindness among diabetics.

Diabetes complications including retinopathy can be reduced or delayed by as much as half with proper diabetic care and routine follow-up. Controlling blood sugar levels and undergoing frequent ocular examinations can help decrease the incidence of diabetic retinopathy. Even with perfect therapeutic considerations, however, it is generally not possible to prevent or moderate diabetic retinal problems. Making the general public aware is a critical step in building a successful program to combat any disease in the community, and this is especially true for the growing problem of diabetic retinopathy. It is impossible for anyone to aid the cause of preventing vision impairment in diabetics without first becoming aware of the disease [10].

In this cross-sectional study, more than two-thirds of diabetic patients knew that diabetes mellitus may lead to eye problems. This finding is slightly lower than that reported by other studies in Saudi Arabia (75.6%), Oman (72.0%), and Australia (96.0%) [10-12]. However, this knowledge was better than the findings reported by studies from India (50.0%) and the United States (52.0%) [13,14]. This disparity between the findings of the Indian and American studies and the current study may be related to the difference between the participants. While this study included diabetics the other two studies included non-diabetics who might not be interested in knowing about diabetes mellitus or its complications.

In this study, 72.5% of diabetics knew that they should undergo regular eye examinations, which was almost similar to the results of studies conducted in Saudi Arabia and Australia (73.8% and 75.0%, respectively [15,16]. On the other hand, this knowledge was inadequate among the Omani population [11]. Rani et al. described that 36.5% of diabetic patients who knew about diabetic retinopathy decided that there was no need to seek the advice of an ophthalmologist whenever their blood sugar was controlled [15]. On the other hand, in this study, 64.5% of diabetic patients denied the importance of visiting an ophthalmologist with controlled blood glucose levels. This finding is concerning because according to clinical guidelines, all diabetic patients should undergo regular annual fundus examinations. Therefore, healthcare providers should stress the need for diabetic patients to be adherent to their regular annual fundus examinations.

Unfortunately, 30.3% and 21.5% of diabetic patients ignored the role of surgery and adequate diabetes mellitus control in the management of diabetic retinopathy. Similar to the findings from a Syrian study in which 34% of respondents stated that surgery or strong diabetes mellitus control are not good treatment options for diabetic retinopathy [16]. These findings reflect the poor knowledge level among diabetic patients about the most valuable measures of diabetic retinopathy management and trigger researchers to propose clear recommendations to overcome this weakness in management. Patients’ concerns about diabetic retinopathy management would have a great impact on their commitment to controlling their blood glucose levels, which, in turn, will improve their eye health and protect it from diabetic retinopathy.

Surprisingly, 57.0% of diabetic patients had undergone regular ocular examinations but the frequencies of these regular examinations were 4.0% monthly, 16.7% once every six months, and 36.3% one yearly examination. This observation was poor in comparison with the results of another Saudi study in AlJouf and Hail province where 95.0% of diabetic patients conducted ocular examinations, 12.1% went for monthly check-ups, 33.9% once in six months, and 49.0% once yearly [10]. Mwangi et al. reported that only 50% of diabetic patients underwent ocular examinations, with a frequency of 27% once yearly, 10% once every six months, and 17% once monthly [10], whereas Ovenseri-Ogbomo et al. found that 34.6% of their patients had never had their eyes examined and just 19.5% had an eye examination within one year [17]. It is obvious that all these different studies explored the lack of regular eye examinations among diabetic patients which is another critical point to be addressed and handled well according to this study’s recommendations. This can be managed by ensuring that family physicians are adherent to family practice guidelines and refer their diabetic patients for regular fundus examinations in addition to providing continuous education for diabetic patients about the importance of regular eye examinations.

Adequate knowledge score about diabetic retinopathy was significantly higher among diabetic patients aged between 25 and 49 years (52.7%), which was much higher than that reported in a study conducted in Ghana (9.7%) [18]. This outcome is logical as young adults have many opportunities to learn and search for knowledge on different websites in comparison to the elderly. Additionally, they may be more compliant with their regular follow-up visits than the elderly, which, in turn, increases their opportunity to obtain more information from their physicians.

Patients’ level of knowledge regarding diabetic retinopathy was significantly associated with their education level as an adequate level of knowledge was observed among patients who had a university education level (21%). This finding is not unexpected because highly educated patients have much better information about their health status and are keen to know more about their disease and management options. Against this result, an Indian study did not find any significant difference in the level of education and patients’ knowledge [19].

The duration of diabetes is a primarily non-modifiable predictor of the development and progression of diabetic retinopathy [20]. This study reported a significant relationship between the duration of diabetes mellitus and patients’ knowledge level. The majority of patients (60.5%) who had DM for more than 10 years appeared to have an acceptable level of knowledge (p = 0.018). It can be explained by the higher disease duration in association with more exposure to health education from healthcare providers, social media, or even from other patients.

Adequate knowledge levels were found among diabetic patients who were on insulin therapy (55.3%). It may be due to the higher compliance of diabetic patients who are treated by injections and higher follow-up frequency. This explanation was supported by the significant association observed between the level of knowledge and adherence to antidiabetic medications as 92.8% of diabetic patients who were compliant with their treatment had more adequate knowledge about diabetic retinopathy (p = 0.022). This finding is greater than that documented in another Saudi Arabian study (71.1%) [10].

Concerning patients’ source of knowledge, doctors and other diabetic patients were the main sources of knowledge in this study compared to a study from Bangladesh which showed that the two most common sources of information were friends and neighbors (51%) and family (49%) [21]. This result reflects the impact of medical doctors in educating diabetic patients regarding diabetic retinopathy, especially who had adequate knowledge.

This study had some limitations concerning the type of the study which gives a superficial but not an in-depth view of patient knowledge. Therefore, other qualitative studies should be done to understand diabetic patients’ perceptions regarding diabetic retinopathy. This study did not examine other factors that could affect patients’ knowledge about diabetic retinopathy such as whether family physicians followed clinical guidelines regarding follow-up examination for diabetic patients. In addition, the assessment of their perceptions as regards the availability of an adequate referral system when their family physicians refer them to an ophthalmologist for fundus examinations, as well as the response and the time needed to take an appointment. The referral system had to be taken into research consideration, especially in this study setting which has limited healthcare facilities as there are only two general hospitals that delay referrals for fundus examination for months until taking an appointment in one of them. Distributing the questionnaires on social networks is also a limitation because we could not control the reliability of the information of the persons concerned.

The key strengths of this study can be summarized in two points. First, the investigation of the awareness level about diabetic retinopathy among a vulnerable group of patients in a remote area like Al Qunfudah which has limited health facilities. Hence, this study can become an initiative to improve awareness levels among both healthcare providers and patients to improve their illness management and prevent the incidence of diabetic retinopathy. Second, choosing the sample through a random sampling technique limited selection bias. Moreover, the snowball sampling method helped the data collectors to reach many diabetic patients who could not be met inside the PHCs.

## Conclusions

Diabetic patients had average knowledge about diabetic retinopathy but little awareness regarding the frequency of regular eye examinations and the physicians who should conduct them. Age, educational level, duration of diabetes mellitus, types of antidiabetic medications, degree of adherence to these medications, and source of information were determinants for the level of knowledge about diabetic retinopathy among diabetic patients in Al Qunfudah district, Kingdom of Saudi Arabia.

In light of this study’s findings, it is recommended to ensure continued effective communication between family physicians and other healthcare providers and diabetic patients regarding their illness and its complications. Preventive plans should be developed regarding diabetic retinopathy and regular eye examinations. Media contribution as a source of participants’ knowledge in this study was low; therefore, media, both broadcast and television programs, should be launched to increase the level of awareness not only among diabetic patients but also among the general population to improve their awareness and practice toward eye care and prevent visual impairment among diabetic individuals and the community.

## References

[1] (2016). Worldwide trends in diabetes since 1980: a pooled analysis of 751 population-based studies with 4.4 million participants. Lancet.

[2] Alharbi AM, Alhazmi AM (2020). Prevalence, risk factors, and patient awareness of diabetic retinopathy in Saudi Arabia: a review of the literature. Cureus.

[3] Galicia-Garcia U, Benito-Vicente A, Jebari S (2020). Pathophysiology of type 2 diabetes mellitus. Int J Mol Sci.

[4] (1991). UK Prospective Diabetes Study (UKPDS). VIII. Study design, progress and performance. Diabetologia.

[5] Lee R, Wong TY, Sabanayagam C (2015). Epidemiology of diabetic retinopathy, diabetic macular edema and related vision loss. Eye Vis (Lond).

[6] Scanlon PH, Aldington SJ, Stratton IM (2013). Epidemiological issues in diabetic retinopathy. Middle East Afr J Ophthalmol.

[7] AlHargan MH, AlBaker KM, AlFadhel AA, AlGhamdi MA, AlMuammar SM, AlDawood HA (2019). Awareness, knowledge, and practices related to diabetic retinopathy among diabetic patients in primary healthcare centers at Riyadh, Saudi Arabia. J Family Med Prim Care.

[8] Alzahrani SH, Bakarman MA, Alqahtani SM (2018). Awareness of diabetic retinopathy among people with diabetes in Jeddah, Saudi Arabia. Ther Adv Endocrinol Metab.

[9] Ahmed RA, Khalil SN, Al-Qahtani MA (2016). Diabetic retinopathy and the associated risk factors in diabetes type 2 patients in Abha, Saudi Arabia. J Family Community Med.

[10] Al Zarea BK (2016). Knowledge, attitude and practice of diabetic retinopathy amongst the diabetic patients of AlJouf and Hail province of Saudi Arabia. J Clin Diagn Res.

[11] Khandekar R, Harby SA, Harthy HA, Lawatti JA (2010). Knowledge, attitude and practice regarding eye complications and care among Omani persons with diabetes - a cross sectional study. Oman J Ophthalmol.

[12] Schmid KL, Schmid LM, Pedersen C (2003). Knowledge of the ocular effects of diabetes among the general population of Australia and the members of Diabetes Australia. Clin Exp Optom.

[13] Namperumalsamy P, Kim R, Kaliaperumal K, Sekar A, Karthika A, Nirmalan PK (2004). A pilot study on awareness of diabetic retinopathy among non-medical persons in South India. The challenge for eye care programmes in the region. Indian J Ophthalmol.

[14] Muñoz B, O'Leary M, Fonseca-Becker F (2008). Knowledge of diabetic eye disease and vision care guidelines among Hispanic individuals in Baltimore with and without diabetes. Arch Ophthalmol.

[15] Rani PK, Raman R, Subramani S, Perumal G, Kumaramanickavel G, Sharma T (2008). Knowledge of diabetes and diabetic retinopathy among rural populations in India, and the influence of knowledge of diabetic retinopathy on attitude and practice. Rural Remote Health.

[16] Hamzeh A, Almhanni G, Aljaber Y (2019). Awareness of diabetes and diabetic retinopathy among a group of diabetic patients in main public hospitals in Damascus, Syria during the Syrian crisis. BMC Health Serv Res.

[17] Mwangi MW, Githinji GG, Githinji FW (2011). Knowledge and awareness of diabetic retinopathy amongst diabetic patients in Kenyatta National Hospital, Kenya. Int J Humanit Soc Sci.

[18] Ovenseri-Ogbomo GO, Abokyi S, Koffuor GA, Abokyi E (2013). Knowledge of diabetes and its associated ocular manifestations by diabetic patients: a study at Korle-Bu Teaching Hospital, Ghana. Niger Med J.

[19] Foster T, Mowatt L, Mullings J (2016). Knowledge, beliefs and practices of patients with diabetic retinopathy at the University Hospital of the West Indies, Jamaica. J Community Health.

[20] Klein R, Klein BE, Moss SE, Davis MD, DeMets DL (1989). The Wisconsin Epidemiologic Study of Diabetic Retinopathy. IX. Four-year incidence and progression of diabetic retinopathy when age at diagnosis is less than 30 years. Arch Ophthalmol.

[21] Mumu SJ, Saleh F, Ara F, Haque MR, Ali L (2014). Awareness regarding risk factors of type 2 diabetes among individuals attending a tertiary-care hospital in Bangladesh: a cross-sectional study. BMC Res Notes.

